# Longitudinal Associations of Dietary Fiber Intake with Glycated Hemoglobin and Estimated Insulin Sensitivity in Adults with and without Type 1 Diabetes

**DOI:** 10.3390/nu15214620

**Published:** 2023-10-31

**Authors:** Arpita Basu, Andrew Hooyman, Leigh Ann Richardson, Amy C. Alman, Janet K. Snell-Bergeon

**Affiliations:** 1 Department of Kinesiology and Nutrition Sciences, School of Integrated Health Sciences, University of Nevada Las Vegas, Las Vegas, NV 89154, USA; andrew.hooyman@unlv.edu; 2 School of Biological Health Systems Engineering, Arizona State University, Tempe, AZ 85281, USA; 3 Department of Epidemiology and Biostatistics, School of Public Health, University of Nevada Las Vegas, Las Vegas, NV 89154, USA; richal7@unlv.nevada.edu; 4 College of Public Health, University of South Florida, Tampa, FL 33620, USA; aalman@usf.edu; 5 Barbara Davis Center for Diabetes, Anschutz Medical Campus, University of Colorado, Aurora, CO 80045, USA; janet.snell-bergeon@cuanschutz.edu

**Keywords:** dietary fiber, glycated hemoglobin, estimated insulin sensitivity, prediabetes, prevention

## Abstract

Dietary fiber, an essential bioactive compound in plant-based diets, is of public health concern based on habitual low intakes in the general population. Not much data are available on how habitual dietary fiber is associated with glycemic control in type 1 diabetes (T1D) as well as in prediabetes and normoglycemic adults. To address this gap, we conducted a six-year longitudinal analysis of an original cohort in adults with and without T1D (*n* = 1255; T1D: *n* = 563; non-diabetes mellitus (non-DM): *n* = 692). Dietary data were collected from a validated food frequency questionnaire, biochemical measures were obtained after an overnight fast, and anthropometric measurements were collected at baseline as well as after three and six years for the follow-up study. Glycated hemoglobin (HbA1c) and estimated insulin sensitivity (eIS) were the main outcomes examined. In adjusted analyses, dietary fiber intake was inversely associated with HbA1c in a minimally adjusted model, but it was positively associated with eIS in a model involving all relevant covariates in non-DM adults. These associations were not significant in the T1D group. Furthermore, when examined by HbA1c cut-offs for glycemic control, an inverse association with dietary fiber was only observed in adults with prediabetes (all *p* < 0.05). At a six-year mean (±SD) dietary fiber intake of 17.4 ± 8.8 g for non-DM and 17.0 ± 8.2 g for the T1D group, protective associations against poor glycemic control were observed in those without diabetes and in prediabetes.

## 1. Introduction

Type 1 diabetes (T1D), a global public health burden primarily resulting from the immune-mediated destruction of pancreatic beta-cells and insulin deficiency, has been increasing globally with the highest rates in northern Europe (age-standardized prevalence of 4.4/1000 per year) and the lowest in Asia (age-standardized prevalence of 0.6/1000 per year) [1]. While several factors are being investigated in its etiology, dietary factors have been shown to play a key role in the epigenetic modifications that lead to pancreatic beta cell failure [2,3,4]. Prediabetes, a risk factor for type 2 diabetes (T2D), has been growing globally, and much of this has been fueled by poor dietary intakes and sedentary lifestyle factors promoting weight gain and insulin resistance [5]. Dietary fiber has been widely recognized for its beneficial role in improving glycemic control and cardiometabolic risks in multiple randomized controlled trials including participants with and without diabetes [6,7,8]. Prospective cohort studies show high dietary fiber intake, classified as >25 g of total daily fiber intake for women and >38 g of total daily fiber intake for men, to be significantly associated with reduced risks of type 2 diabetes (T2D) [9]. In a systematic review and meta-analysis of 42 clinical trials including adults with prediabetes and diabetes, the researchers concluded that increasing daily fiber intake by 15 g or up to 35 g could decrease risks of premature mortality in diabetes [10]. Supported by these data, the American Diabetes Association (ADA) recommends a daily fiber intake of 25 g/day for women and 38 g/day for men as a component of effective dietary management of diabetes [11]. Dietary fiber has multiple mechanisms that may lead to reduced risks of developing diabetes and/or maintaining optimal glycemic control as follows: increasing satiety and leading to weight loss and improved insulin resistance; decreasing postprandial glucose excursions; and enriching the gut microbiome, leading to the production of short-chain fatty acids and lower systemic inflammation [9,12]. While these physiological functions of dietary fiber justify its increasing consumption in diabetes prevention and management, reported observational data do not clearly identify its role in glycemic control and insulin sensitivity in context of a habitual diet in adults with and without T1D.

In a cross-sectional study of adults with T1D (*n* = 111), those with increasing fiber intake conforming with ADA recommendations of ≥14 g/1000 kcal exhibited lower systolic and diastolic blood pressure than those with lower fiber intake. However, the study reported no significant associations with glycemic control and insulin dosage [13]. In another cross-sectional analysis of adults with T1D (*n* = 118), those consuming higher dietary fiber intake (25 to 50 g/day) versus those who consumed lower amounts did not have significant differences in glycated hemoglobin (HbA1c) and estimated glucose disposal rate [14]. These null differences can also be explained by the small number of adults who reported consuming high dietary fiber in their studies. In another cross-sectional study of adults with T1D (*n* = 118), evaluation of healthy lifestyle factors (regular physical activity, optimal diet quality including adequate fiber intake, and non-smoking status) did not reveal any significant associations with HbA1c and estimated glucose disposal rate across increasing levels of adherence [15]. In contrast, in a cross-sectional study of youths with T1D (*n* = 252), lower fiber intake was significantly associated with higher HbA1c [16]. In addition to T1D, few observational studies have reported the association of total dietary fiber intake with prediabetes incidence and management. In a prospective cohort study of Chinese adults (*n* = 18,085), total dietary fiber and fruit fiber were inversely associated with the incidence of prediabetes [17]. In another study of adults at high risk of T2D, dietary fiber intake was shown to be inversely related with waist circumference but not with body weight and glycemic status [18]. Thus, associations of dietary fiber with glycemic status appear to be conflicting in adults with T1D as well as in prediabetes in which dietary factors trigger the risk of progression to T2D. In adults without any form of diabetes, the association of dietary fiber intake with glycemic status and insulin resistance is not clear. In a small study of adults without diabetes (*n* = 54), dietary fiber intake at a dose of 7.0 g/1000 kcal or higher was associated with improved postprandial blood glucose [19]. In a prospective cohort study of eight years in the Nurses’ Health Study II (*n* = 13,110) of participants free of diabetes, habitual dietary fiber intake revealed an inverse association with the incidence of gestational diabetes. However, this association was most pronounced when comparing the lowest quintile (mean ± SD: 12.0 ± 1.6 g) vs. the highest quintile (26.3 ± 5.4 g) of habitual dietary fiber intake daily [20].

Clinical trials specific to T1D provide mixed results on their role in glycated hemoglobin and insulin sensitivity in adults and children. In a study of eight adult men with T1D, the addition of guar gum (15 g/day) as a source of fiber for four months revealed no effects on insulin sensitivity and glucose disposal rate following fiber supplementation [21]. A prebiotic fiber supplement of inulin for 12 weeks in children with T1D improved gut microbiome but did not affect glycated hemoglobin and in addition increased C-peptide levels when compared to placebo [22]. In another study of young adults with T1D, habitual total fiber intake (ranging 12 to 14 g/day) increased short-chain fatty acid-producing bacteria, but associations with glycemic control were not reported [23]. Thus, the role of dietary fiber in the context of T1D needs further investigation at habitual intake levels to further design effective interventions in reducing the disease burden.

The Coronary Artery Calcification in Type 1 Diabetes (CACTI) study is an observational prospective cohort to examine the progression of coronary artery calcification as a predictor of atherosclerosis and cardiovascular events, accounting for traditional cardiovascular risks in adults with and without T1D [24,25]. Cross-sectional data from this cohort have previously reported that the consumption of an atherogenic diet is associated with coronary calcification [26] and low fiber intake is associated with poor glycemic control in adults with and/or without T1D [27]. Previous studies on the fiber–glycemia relationship in T1D are few and conflicting; many are cross-sectional in nature, conducted on a small number of T1D adults, and often do not report associations with insulin response. Thus, there is an important need to examine longitudinal associations of dietary fiber intake with established biomarkers of glycemic control in T1D adults as well as in those without diabetes and in prediabetes. In the present report, we aim to examine six-year longitudinal associations of dietary fiber intake with HbA1c and estimated insulin sensitivity in adults with and without T1D. In addition, we also examined how these longitudinal associations differ by different levels of glycemic control, such as normoglycemic, prediabetes and diabetes categories defined at baseline.

## 2. Methods

This report is based on secondary data analyses of the CACTI study with procedures previously published in detail [25,26]. Informed consent was obtained from all study participants, and ethics approval was obtained from the Colorado Multiple Institutional Review Board (ethics code 97-661). The study was registered at clinicaltrials.gov (NCT00005754).

### 2.1. Dietary Data and Glycemic Control

Information on dietary data were collected using a food frequency questionnaire (Harvard 1988) as previously described at baseline, visit 2 (3-year follow-up) and visit 3 (6-year follow-up) of the study [26,28]. The FFQ included plant-derived food groups as major sources of fiber and included the following categories: fruits, vegetables, breads, cereals, starches, baked goods, and miscellaneous categories of fiber including nuts, bran and wheat germ. The total fiber was calculated based on the USDA food composition database. Biochemical variables on blood lipids (LDL cholesterol and triglycerides) and HbA1c were determined in blood samples collected after an overnight fast as previously described [26]. At each visit, body weight, height and waist circumference were also recorded for each participant. Systolic blood pressure (SBP) and fifth-phase diastolic blood pressure (DBP) were measured during the rest state, and an average of three measurements was reported. As an index of insulin sensitivity, eIS was calculated based on a method validated by Duca et al. using a best-fit prediction model based on waist circumference, triglycerides, adiponectin, and diastolic blood pressure [29]. HbA1c was categorized as normal (<5.7%), prediabetes (5.7–6.4%) and those with clinical diagnosis of diabetes (≥6.5%) according to the clinical care guidelines of the ADA at baseline [30]. Physical activity assessment was conducted utilizing the Modifiable Activity Questionnaire [31].

### 2.2. Statistical Analyses

The primary goal of our analyses was to examine the association of the six-year longitudinal intakes of dietary fiber with glycemic control with HbA1c and eIS as outcomes. We also performed an exploratory analysis to examine association of dietary fiber intake with levels of glycemic control (normal, prediabetes and diabetes status defined at baseline). We first summarized the demographics at baseline and year 3 by the diabetes group. The paired *t*-test and McNemar test were applied to measure differences between baseline and year 3 for the non-T1D and T1D groups for continuous and categorical variables, respectively. We mainly used the mixed-effects model to examine our primary goal of the association between dietary fiber intake and glycemic control in combined analysis as well as those stratified by diabetes status. Model adjustments were made as follows: model 1 was adjusted for age, sex, calories, follow-up time, and diabetes status (only for pooled analysis). Model 2 was further adjusted for BMI, and the final model 3 also included LDL-cholesterol and physical activity levels. We also performed a Spearman correlation analysis to examine associations of dietary fiber intake with HbA1c and eIS. Statistical analyses were performed using R version 4.0.3. A two-sided alpha level of 0.05 was used to define statistical significance.

## 3. Results

### 3.1. Baseline and Year 6 Participant Characteristics

**Figure 1:**
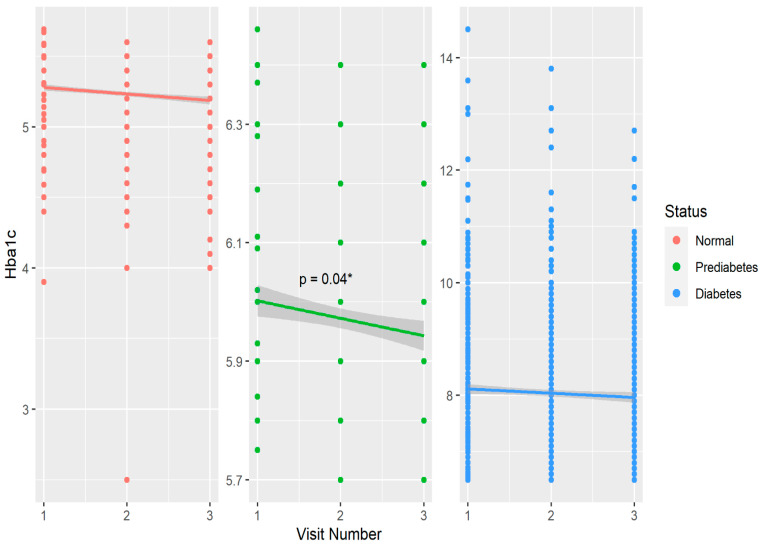
Distribution and changes in glycated hemoglobin over six years stratified by glycemic control in the CACTI study. * *p* < 0.05 over three visits in the prediabetes group.

Table 1 shows baseline and year 6 features of the cohort compared within T1D, prediabetes, and non-T1D groups defined by HbA1c at baseline. Within the non-T1D group, age, BMI, HbA1c, and fiber intake significantly increased over six years, while serum triglycerides and LDL cholesterol decreased during this timeframe. In the prediabetes group, age, HbA1c and fiber intake also increased significantly over six years. Among the T1D participants, similar increases were observed for age, BMI, and fiber over six years. (Table 1). Physical activity and dietary caloric intake did not differ over six years in any group. Regarding biomarkers of glycemic control, HbA1c increased significantly within the non-DM and prediabetes groups over six years, and eIS significantly increased in the prediabetes and T1D groups (Table 1). The six-year mean (±SD) daily dietary fiber intake was 17.4 ± 8.8 g in non-DM, 17 ± 6.3 g in prediabetes, and 17.0 ± 8.2 g in T1D groups. Figure 1 shows the change in glycated hemoglobin over six years in groups stratified by glycemic status.

### 3.2. Longitudinal Six-Year Associations of Dietary Fiber Intake with HbA1c and eIS

As shown in Table 2, a significant association of dietary fiber intake with glycated hemoglobin was observed only in the minimally adjusted model in the non-T1D group; no significant associations were observed in the T1D group over six years. On the other hand, examining associations of dietary fiber intake with estimated insulin sensitivity revealed more significance following adjustments but again mostly in the non-T1D group including in the final model of all covariates.

### 3.3. Longitudinal Six-Year Associations of Dietary Fiber Intake with HbA1c by Glycemic Control

Table 3 shows a six-year association of dietary fiber intake with glycated hemoglobin stratified by different levels of clinically relevant glycemic control defined at baseline. Overall, significant inverse associations were observed in the prediabetic group including in the final model of all covariates. No significance was observed in groups with normal nor in those with clinical diagnosis of diabetes.

In Table 4, we observe a significant inverse correlation of dietary fiber intake with glycated hemoglobin in adults with T1D at both visits as well as a significant positive correlation of dietary fiber intake with insulin sensitivity in non-DM controls at both visits.

## 4. Discussion

In our longitudinal six-year study of adults with and without T1D, habitual fiber intake revealed a significant but modest inverse association with HbA1c and a positive association with estimated insulin sensitivity in adults without diabetes. Adjusting for BMI weakened this association but remained borderline significant in these adults. No such association was observed in adults with T1D. Interestingly, when examined further by glycemic control using HbA1c cut-offs at baseline, dietary fiber showed an inverse association with glycemic control in adults with prediabetes but not among adults with HbA1c in the normal and diabetic range. Of interest, while habitual dietary fiber intake increased significantly over six years within all groups, the lack of association in those with diabetes suggests that intakes at habitual low levels fail to counteract the disease burden over the years.

Habitual dietary fiber intake in US adults has constantly failed to meet the dietary guidelines as revealed by observational data using national and global surveys. A population-based study comparing four countries revealed the lowest compliance to guidelines of dietary fiber intake in the US population (3%) when compared to Ghana (43%), Jamaica (9%) and Seychelles (6%) [32]. In another observational study of US older adults (*n* = 4125), baseline fiber intake (energy-adjusted total mean fiber intake of 16 ± 5 g/day (mean ± SD)) when categorized by quintiles revealed no significant difference in the number of adults with diabetes in the lowest vs highest quintile (<11.5 vs. >21 g/day) [33]. Patterns of dietary fiber intake among non-US populations also reveal low amounts of habitual fiber intake. In an observational study among adults in Korea (*n* = 143,050) analyzing a habitual fiber intake ranging from approximately 3 to 10 g of dietary fiber per day, a 10-year follow-up revealed inverse associations with all-cause mortality including diabetes as well as cardiovascular mortality in these adults [34]. In another observational study of Asian adults in Japan (*n* = 8925), adults consuming the highest amount of dietary fiber vs. the lowest revealed a significantly lower CVD mortality over 24 years of follow-up [35]. Interestingly, in this prospective study, dietary carbohydrates and starches were not associated with CVD mortality, thus highlighting the role of fiber as a major bioactive non-digestible carbohydrate in modulating metabolic risks. In the UK Biobank study of 195,658 adults, a habitual mean fiber intake of about 16.5 g/day revealed a significant non-linear inverse association with all-cause mortality and incident CVD [36]. In more recently reported data from the US NHANES survey (2013–2018; *n* = 14,640), habitual dietary fiber intake was still below optimal levels in groups classified as ‘no diabetes’, ‘prediabetes’, and those with ‘type 2 diabetes’. Habitual fiber intake revealed a significant inverse association with HbA1c only in adults within the normal range of glycemic control but not in the other two groups [37].

Diabetes management emphasizes increasing dietary fiber intake, and several clinical trials have revealed improved glycemic control with fiber supplementation in adults with diabetes who met or exceeded daily recommendations for dietary fiber intake [6,7,10,38]. In more recent years, clinical trials provide evidence on the role of dietary fiber in improving glycemic control, including blood glucose and HbA1c in medicated patients with T2D. Results reveal that the fiber-supplemented group had an increased diversity of bacteria-producing short-chain fatty acids (SCFA) causing improvements in HbA1c by increasing glucagon-like peptide-1 production [39]. Similar associations of dietary fiber intake with SCFA-producing bacteria have also been reported in young adults with T1D [23]. In a systematic review and meta-analysis of nine clinical trials in adults with T2D, dietary fiber supplementation decreased HbA1c and increased beneficial bacteria that produce SCFA and also specific bacteria that increase glucose transporter activities and decrease hepatic gluconeogenesis [40]. These mechanisms explain our observations of significant inverse associations with HbA1c and positive associations with insulin sensitivity despite low levels of habitual intakes in adults without diabetes and those with prediabetes. Lifestyle interventions including dietary modifications have been shown to reverse/slow the progression of prediabetes to diabetes [41,42,43]; our findings provide evidence on this association in the context of dietary fiber. Collectively, these data suggest the need for health-care providers to emphasize increasing dietary fiber intake in adults preferably through supplemental fiber intake to the otherwise low habitual fiber diet. Such a strategy to focus on a single component, such as fiber, could be more effective in improving HbA1c than making multiple dietary changes in adults with diabetes. The significant inverse association of habitual dietary fiber intake with glycated hemoglobin only in the prediabetes group (but not in those with normal) as well as HbA1c values that fall in the clinical diagnosis of diabetes is clinically important. These observations further add to the existing studies on the inverse association of dietary fiber with diabetes incidence in prospective studies. The mechanisms by which dietary fiber may promote insulin sensitivity and lower blood glucose have been largely understood as the role of dietary fiber in preventing weight gain and altering hepatic gene expressions that promote fat oxidation and increase insulin sensitivity [6]. Emerging research studies increasingly point out the role of the gut microbiome in producing metabolites that either promote or inhibit insulin sensitivity, and one of the reported pathways has been an increase in branched chain amino acids (BCAAs) by the gut microbiome that cause insulin resistance [44]. In a previously reported study from our group, dietary whole strawberries consumed at a dose of 2.5 servings for four weeks providing a combination of fiber with various polyphenols and vitamins that led to significantly reduced BCAAs in adults with prediabetes [45]. Thus, fiber in fruits and vegetables is indeed one of the bioactive compounds promoting insulin sensitivity as observed in our current study. Resistant starch, resulting from the cooling and retrogradation of cooked starch, leads to an indigestible form of starch that has been shown to improve postprandial glycemia in adults with T1D [46,47]. This certainly deserves attention in future trials. Furthermore, many of these effects are dependent on the quality of fiber, its role in delaying gastric emptying and forming a gel-like substance within the intestinal content as well as affecting the gut hormones, which all contribute to glucose absorption and optimal glycemic control [9,48]. While the beneficial effects of fiber are understood to be mediated by these mechanisms, there is a possibility that such mechanisms may not work similarly in all three groups: namely, the T1D, prediabetes and non-diabetic controls in our study. Physiological differences, such as the administration of insulin in the T1D group, and the independent effects of insulin in delaying gastric emptying [49,50] may have masked the benefits of habitual dietary fiber on glycemic control. Similarly, at observed levels of intakes, dietary fiber may not be enough to further improve normoglycemia in non-diabetic adults, thus explaining our observed significance of dietary fiber only in the prediabetic group not on insulin therapy.

An estimated insulin sensitivity index was developed based on waist circumference, serum triglycerides, adiponectin, and systolic blood pressure, and it has been previously shown to be inversely associated with albuminuria in the CACTI study [51]. Adiponectin, an anti-inflammatory cytokine, has been positively associated with insulin sensitivity, mainly via serving as a downstream effector molecule leading to insulin receptor activity [52]. Along these observations, a high-fat diet-induced weight gain and elevated serum and liver triglyceride levels have been shown to reduce insulin sensitivity; adiponectin reverses this process [52]. The treatment of obese animals with adiponectin suppressed endogenous glucose production as well as decreased liver and muscle plasma membrane triglyceride content that led to improved insulin receptor activity and thus insulin sensitivity [53]. Elevated systolic blood pressure or hypertension has also been shown to be an outcome of insulin resistance, as insulin has been shown to exert a peripheral vasodilatory effect by stimulating the production of endothelial nitric oxide and improving arterial stiffness [54]. Consequently, a high-fiber diet has been shown to lower serum triglycerides and systolic blood pressure, adiposity and inflammation [9], all of which lead to improved insulin sensitivity and a reduced risk of developing diabetes and its complications. Interestingly, in our study, insulin sensitivity in those with prediabetes as well as with T1D significantly increased over six years with concomitant increases in dietary fiber. However, the association was significant only in the prediabetes group but not in the T1D group, which emphasizes the need for further dietary improvements to counteract the disease burden over years.

However, few studies have used this model of estimated insulin sensitivity to examine associations with dietary factors in diabetes. In general, weight loss diets and plant-based diets have been shown to increase insulin sensitivity in adults [55]. Furthermore, exercise has been shown to stimulate insulin sensitivity more than dietary changes in healthy adults [56,57]. Again, the homeostatic model of insulin resistance requires glucose and insulin levels in the fasting state that could be challenging to obtain in large trials for multiple visits. Dietary studies on insulin metabolism in adults with T1D are limited and yield conflicting results on insulin resistance. A high-fiber diet supplementation (15 g/day) for four months in eight adults with T1D did not affect insulin sensitivity and glucose levels [21]. In the Diabetes Control and Complications Trial, one of the largest trials investigating T1D, dietary fiber intake also did not have any significant associations with HbA1c following a five-year follow-up [58]. On the other hand, a low-fat diet for three months improved insulin sensitivity in adults with T1D (N = 10) [59]. Our previous cross-sectional report shows higher dietary manganese intake to be positively associated with estimated insulin sensitivity in T1D adults as well as in non-diabetic controls [60]. Thus, multiple dietary factors may be required to improve insulin sensitivity, and this requires further dose and types of diets examination in clinical trials in adults with established T1D.

Our study has certain limitations which must be taken into consideration for a proper interpretation of the results. First, we determined the total dietary fiber intake and did not categorize by types such as soluble and insoluble fiber that may have a differential impact on glycemic control. Second, FFQ-derived data are prone to recall bias, but repeated data collection over three time points increases the validity of the exposure variables. Third, we did not measure variables that may explain the mechanisms of dietary fiber intake in affecting glycemic control, such as the gut microbiome and serum metabolomics that must be examined in future studies. Fourth, our study examined a six-year association, and a longer follow-up duration may be needed to detect a greater difference in fiber–glycemic control association than what was observed in our study. Finally, our findings lack generalizability to other populations, such as those with type 2 diabetes and its complications. Despite these limitations, our longitudinal analyses reveal important findings that strengthen the evidence for increasing dietary fiber in diabetes prevention and management. Future studies with more precisely monitored means of intake of fiber quantity and components administration are warranted to further substantiate the current findings.

In conclusion, our six-year analyses revealed that habitual mean dietary fiber intake in adults with and without diabetes was below the recommended levels, and overall, it revealed inverse associations with glycated hemoglobin and positive association with insulin sensitivity in adults without diabetes. When further examined by levels of glycemic control, a significant inverse association with glycated hemoglobin was observed only in adults with prediabetes. Furthermore, the positive association of dietary fiber with insulin sensitivity in non-diabetic adults and inverse association with glycated hemoglobin in adults with prediabetes remained robust in the final model that also adjusted for physical activity level, which is an important modulator of insulin sensitivity and glucose transport. Thus, maintaining fiber intake at the observed levels, and increasing dietary fiber intake must be emphasized in the nutrition therapy for diabetes prevention and insulin management.

## Figures and Tables

**Table 1:** Participant characteristics.

	Non-DM					Pre-DM					T1D				
	Baseline (N = 590)		Visit 3 (N = 480)			Baseline (N = 212)		Visit 3 (N = 180)			Baseline (N = 603)		Visit 3 (N = 503)		
Variables	Count	%	Count	%	*p*-value ^†^	Count	%	Count	%	*p*-value ^†^	Count	%	Count	%	*p*-value ^†^
Sex (Female)	318	54	243	51	0.39	81	38	71	39	0.78	297	49	238	47	0.86
	Median	IQR	Median	IQR		Median	IQR	Median	IQR		Median	IQR	Median	IQR	
Triglycerides, mg/dL	98	(73–141)	90	(64–134)	**0.0002**	103	(74–162)	88	(64–132)	**0.001**	79	(62–109)	64	(49–93)	**<0.0001**
Physical Activity, min/week	110	(0–240)	100	(0–263)	0.96	80	(0–270)	90	(0–240)	0.61	60	(0–240)	30	(0–243)	0.35
	Mean	SD	Mean	SD		Mean	SD	Mean	SD		Mean	SD	Mean	SD	
Age, y	39	9	45	9	**<0.0001**	43	8	50	8	**<0.0001**	37	9	44	9	**<0.0001**
BMI, kg/m^2^	26.0	5.0	26.0	5.2	**<0.0001**	27.0	5.0	27.0	5.0	0.14	26.0	4.0	27.0	5.0	**<0.0001**
Calories, kcal/day	1831	635	1866	636	0.17	1829	663	1889	679	0.08	1841	752	1881	708	0.325
LDL, mg/dL	112	33	106	31	**<0.0001**	119	37	106	36	**<0.0001**	101	29	88	31	**<0.0001**
HbA1c, %	5.3	0.3	5.4	0.5	**<0.0001**	6.0	0.2	6.1	0.7	**0.04**	8.1	1.1	8.0	1.2	0.11
Fiber, g/day	17.5	8.8	19.6	10.3	**<0.0001**	16.3	7.0	18.1	8.6	**0.003**	17.3	8.9	18.5	9.4	**0.009**
eIS	16.5	8.3	16.4	8.1	0.74	9.1	4.8	10.4	6.0	**<0.0001**	6.1	3.0	6.6	3.5	**0.0002**

Abbreviations: DM = Diabetes Mellitus; SD = Standard Deviation; IQR = Interquartile Range; BMI = Body Mass Index; LDL = Low-Density Lipoprotein; HbA1c = Hemoglobin A1C; eIS = Estimated Insulin Sensitivity; ^†^ *p*-values were computed by McNemar test for categorical variables and paired *t*-test for continuous variables. *p* < 0.05 in bold font.

**Table 2:** Longitudinal six-year associations of dietary fiber intake with glycated hemoglobin and estimated insulin sensitivity by diabetes status.

Model (M)	Pooled			Non-DM Group			T1D Group		
	Parameter Estimate	95% CI	*p*-Value	Parameter Estimate	95% CI	*p*-Value	Parameter Estimate	95% CI	*p*-Value
Glycated Hemoglobin									
M1	0.0006	−0.0008, 0.002	0.42	−0.003	−0.007, −0.0006	**0.02**	−0.002	−0.004, 0.0007	0.15
M2	0.0007	−0.0007, 0.002	0.33	−0.003	−0.006, 0.0003	0.07	−0.002	−0.004, 0.0007	0.17
M3	0.0006	−0.0007, 0.002	0.38	−0.003	−0.006, 0.0007	0.12	−0.002	−0.004, 0.0003	0.09
Estimated insulin sensitivity									
M1	0.060	0.02, 0.09	**<0.001**	0.09	0.04, 0.14	**<0.001**	0.004	−0.02, 0.03	0.79
M2	0.030	0.002, 0.05	0.06	0.04	0.0004, 0.08	0.05	−0.005	−0.03, 0.02	0.68
M3	0.026	0.00001, 0.05	**0.049**	0.04	0.004, 0.08	**0.03**	−0.008	−0.03, 0.02	0.51

M1: Age, Sex, Calories, Visit (follow-up time), (+Diabetes Status for Pooled Analysis); M2: Model 1 + BMI; M3: Model 2 + LDL + Physical Activity Levels; *p* < 0.05 in bold font.

**Table 3:** Longitudinal six-year associations of dietary fiber intake with glycated hemoglobin by normal, prediabetes and diabetes status defined at baseline.

Model (M)	Normal (HbA1c: <5.7)			Prediabetes (HbA1c: 5.7–6.4%)			T1D (HbA1c: ≥6.5)		
	Parameter Estimate	95% CI	*p*-Value	Parameter Estimate	95% CI	*p*-Value	Parameter Estimate	95% CI	*p*-Value
M1	−0.0009	−0.003, 0.002	0.44	−0.003	−0.005, −0.0002	**0.04**	−0.0007	−0.003, 0.002	0.57
M2	−0.0009	−0.0003, 0.002	0.49	−0.002	−0.005, 0.0003	0.08	−0.0005	−0.003, 0.002	0.62
M3	−0.0005	−0.003, 0.002	0.69	−0.003	−0.005, −0.0001	**0.04**	−0.0008	−0.003, 0.002	0.50

M1: Age, Sex, Calories, Visit (follow-up time), (+Diabetes Status for Pooled Analysis) + total calories; M2: Model 1 + BMI; M3: Model 2 + LDL + Physical Activity Levels; *p* < 0.05 in bold font.

**Table 4:** Spearman correlation analyses by visit (adjusted by age, sex, and calories) for dietary fiber intake with glycated hemoglobin (HbA1c) and estimated insulin sensitivity (eIS).

Covariates		Pooled	Non-DM	T1D
Visit 1	HbA1c	r = −0.01 *p*-value = 0.724	**r = −0.08** ***p*-value = 0.044**	**r = −0.10** ***p*-value = 0.020**
	eIS	**r = 0.07** ***p*-value = 0.011**	**r = 0.19** ***p*-value = <0.0001**	**r = 0.10** ***p*-value = 0.023**
Visit 3	HbA1c	**r = −0.07** ***p*-value = 0.031**	r = −0.04 *p*-value = 0.404	**r = −0.11** ***p*-value = 0.030**
	eIS	**r = 0.13** ***p*-value = 0.0001**	**r = 0.19** ***p*-value = <0.0001**	r = 0.05 *p*-value = 0.308

*p* < 0.05 in bold font.

## Data Availability

The data are not publicly available due to patient privacy.
